# Ameliorative effect of biofabricated ZnO nanoparticles of *Trianthema portulacastrum* Linn. on dermal wounds *via* removal of oxidative stress and inflammation

**DOI:** 10.1039/c8ra03500h

**Published:** 2018-06-13

**Authors:** Ekta Yadav, Deepika Singh, Pankajkumar Yadav, Amita Verma

**Affiliations:** Bioorganic & Medicinal Chemistry Research Laboratory, Department of Pharmaceutical Sciences, Sam Higginbottom University of Agriculture, Technology & Sciences (SHUATS) Allahabad 211007 India amitaverma.dr@gmail.com; Pharmaceutics Laboratory, Department of Pharmaceutical Sciences, Sam Higginbottom University of Agriculture, Technology & Sciences (SHUATS) Allahabad-211007 India pypharm@gmail.com

## Abstract

An impediment in the process of wound healing can be attributed to reactive oxygen species and inflammation. The curative efficacy of green synthesized *Trianthema portulacastrum* Linn. zinc oxide nanoparticles (ZnOTP) was investigated in the present study for evaluation of their wound healing potential in rodents. Total phenolic and flavonoid content of ZnOTP was determined, and antioxidant potential was evaluated by the DPPH method. *In vitro* anti-inflammatory activity of ZnOTP was evaluated by membrane stabilization and albumin denaturation, along with proteinase inhibitory assays. The synthesized ZnOTP were characterized by UV-Visible spectroscopy, Fourier transform infrared (FT-IR) studies, Field Emission Scanning Electron Microscopy (FESEM) and Energy Dispersive X-ray (EDX) studies. The wound healing potential of ZnOTP was monitored by excision and incision wound models. Analyses confirmed the formation of spherical nanoparticles of 10–20 nm size along with strong signals of zinc and oxygen atoms. Significant results (*p* < 0.05) of wound contraction rate, epithelialization and histopathology of the healed tissues of rats confirmed the promising wound healing property of ZnOTP. In addition, inflammatory markers, biochemical estimation such as the hydroxyproline content of granulation tissue, and the profile of antioxidant enzymes also supported the wound healing potential of ZnOTP. The present study advocated the attenuation of wounds *via* antioxidant and anti-inflammatory activities of a green synthesized nano-ointment.

## Abstract 2

The curative efficacy of a green synthesized zinc oxide nano-ointment on wound healing was investigated.

## Graphical abstract

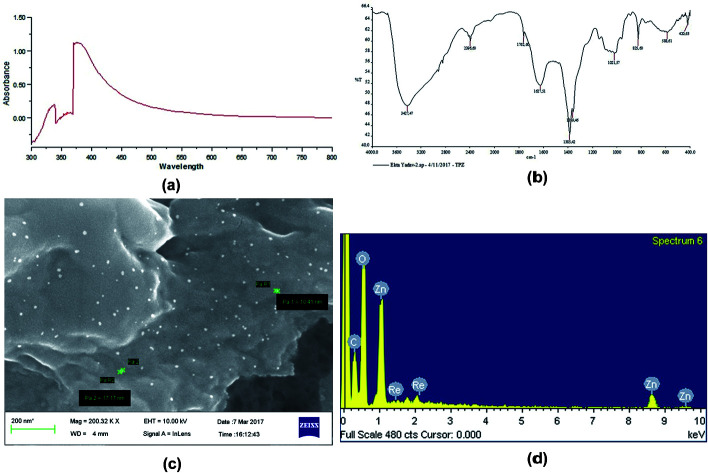

## Introduction

Healing of wounds and tissue repairing are intricate, dynamic and systematic processes which are associated with the reformation and anatomical continuum and function of injured tissues. The process of wound healing is strictly controlled by various growth factors and cytokines are released at the injury site. Essentially three overlying phases, *i.e.* inflammation, proliferation and remodeling are involved in all wound repairing processes.^1^ Functions of these three phases occur in proper co-ordination and persist for a definite time period with suitable intensity.^2^ Therefore, at the onset of the inflammation phase, different inflammatory cells such as neutrophils, macrophages, fibroblasts and endothelial cells lead to the production of reactive oxygen species (ROS) at the wound region, ultimately resulting in the prevention of responsible mechanisms for consequent phases of the wound healing process (granulation and remodeling), and this causes a delay in the healing of the wound.^3^ Oxidative stress induced damage of tissue leads to an imbalance of pro-oxidants and antioxidants. Therefore, a wound region applied with an ointment having antioxidant potential on the wound surface is used for the normal process of wound healing in clinical as well as experimental medicinal formulations.^4^ Furthermore, the antioxidant approach also supports the reduction of inflammation at an early stage, which directly encourages regenerative processes at the later stage of wound healing.^5^

Nowadays nanoparticles, as wonders of medicine, are envisaged as a more impactful and rapidly developing technology in several other fields of human life as well, *i.e.* optics, chemical sensors, electronics, solar cell storage, food packaging and cosmetics, all over the world.^6,7^ Nanoparticles are deemed more impressive as they have unique physical properties, *i.e.* relatively small size, higher surface energy, and optical, magnetic, chemical and biological behaviors.^8,9^

Different types of metal oxide nanoparticles, *i.e.* silver, ferric, zinc, titanium and copper oxides, have been known to have strong antimicrobial properties. Among various metal oxide nanoparticles, zinc oxide nanoparticles (ZnONP) are widely utilized for biological applications such as antibacterial,^10^ antifungal, anti-inflammatory^11^ and as wound healing agents *via* the promotion of fibroblast proliferation and angiogenesis^12^ due to the unique properties of chemical stability and biocompatibility of the nanoparticles. ZnO has been approved as safe for use by the U.S. Food and Drug Administration.^10^ It has been reported that Zn ions released from ZnO promote migration of keratinocytes towards the wound area and accelerate the wound healing process.^13^ Moreover, for dermatological purposes, Zn has sufficient ability to act as a safe antimicrobial, anti-inflammatory and wound repairing agent.^14^ Topical application of Zn is demonstrated to be superior to oral administration as it possesses a potency for minimizing super infections and necrotic material through promotion of the local defense system and the collagenolytic effect. Additionally, the sustained release of Zn ions encourages epithelialization of wounds.^15^

In recent years, a green synthesis method for nanoparticle fabrication has attracted more attention from researchers over traditional chemical methods as it does not need any additional capping agent which leads to the formation and absorption of several toxic chemicals on the surface, ultimately altering the therapeutic application.^16,17^ Synthetic ZnONP at high concentrations mediate cytotoxicity by generation of ROS.^18^ The existence of various phytochemicals, acting as reducing agents, may suppress ROS production.^19^ Therefore, there is a worthwhile interest in integrating zinc oxide (ZnO) with dermal biomaterials.^10,20^ Biofabrication of plant extract mediated ZnONP through a co-precipitation method is considered to be a nontoxic, cost effective, ecofriendly and simple process.^21^

*Trianthema portulacastrum* Linn. (TP), a traditional and dietary plant belonging to the Aizoaceae family, is indigenous to Southeast Africa, tropical America and Asia.^22,23^ TP is capable of growing in sunny desert areas including Arizona and also grows abundantly as a “weed” in well irrigated and high rainfall areas, particularly in India and neighboring countries, *i.e.* Bangladesh, Pakistan and Sri Lanka. It is used as a valuable herb in the Indian traditional medicinal system, such as in Ayurvedic medicine.^24,25^ TP has been reported to possess different types of bioactive chemical constituents such as alkaloids, glycosides, and phenolic and flavonoid compounds. TP has been utilized as an antioxidant and as hepatoprotective, antifungal, anti-inflammatory and wound repairing agents, *etc.*^26^

Considering the anti-bacterial, anti-inflammatory, dermal fibroblastic and angiogenesis accelerator properties of ZnONP, and the wound healing activity of TP, the present study aimed to synthesize *via* green methods, characterize and evaluate the dermal wound healing potential of ZnO nanoparticles of TP aqueous extract (ZnOTP) through excision and incision wound models in rodents along with provide an assessment of the possible mechanism by the analysis of the anti-oxidant enzyme profile, evaluation of anti-inflammatory biomarkers and biochemical estimation.

## Material and methods

### Chemicals and reagents

Sodium chloride, sodium hydroxide, sodium azide, aluminum chloride, hydrogen peroxide, hydrochloric acid, acetic acid, trichloroacetic acid and zinc acetate were purchased from Qualigens (Mumbai, India). Folin and Ciocalteu’s phenol reagent and bovine serum albumin were obtained from Merck KGaA (Darmstadt, Germany). Nitro blue tetrazolium (NBT) and ethylene diamine tetra acetic acid (EDTA) were procured from Hi Media Laboratories Pvt. Ltd., Mumbai, India. Rutin, gallic acid, 1,1-diphenyl-2-picrylhydrazyl (DPPH), azocasein and 5,5′-dithio-bis-2-nitrobenzoic acid (DTNB) were purchased from Sigma-Aldrich (St. Louis, Missouri, United States). All chemicals were of analytical and HPLC grade.

### Collection and authentication of plant

Fresh leaves of TP were collected from a rural area of the Rewari district, Haryana, India. Plant material was subjected to scientific identification and authentication by Prof. R. M. Kadam, Head of Department, Department of Botany, Mahatma Gandhi Mahavidyalaya, Latur, Maharastra, India.

### Preparation of plant extract

About 10 g of air dried plant leaves was added into a 500 mL Erlenmeyer flask followed by the addition of 250 mL distilled water and heating at 80 °C for 1 hour with continuous stirring at 200 rpm. After cooling the resultant solution, it was filtered using Whatman filter paper no. 1. The filtrate was marked as aqueous extract and preserved in a refrigerator for further study.

### Green synthesis of ZnOTP

The synthesis of ZnOTP started with the addition of zinc acetate (1 mM) to 50 mL Milli-Q water and this was placed on a stirrer for 60 minutes. Then, 20 mL of NaOH solution was added slowly into the resulting zinc acetate solution followed by the pouring of 25 mL plant extract into the solution. The resultant reaction mixture was incubated for 60 minutes and it was then left to stir for 3 hours. A change in color (yellow) of the solution confirmed ZnONP formation. The separation of precipitates was performed with centrifugation of the reaction mixture at 60 °C for 15 minutes at 8000 rpm and the pellet was collected. The pellet was dried in a hot air oven at 80 °C for 2 h and was preserved in air-tight bottles for further studies.^9^

### Characterization of ZnOTP

#### UV spectrophotometry

ZnOTP were subjected to analysis by a UV-visible spectrophotometer (UV-2450, Shimadzu) to determine their optical characteristics. A synthesized sample was re-suspended in sterile de-ionized water and scanned.

#### Fourier transform infrared (FT-IR) spectroscopy

An FT-IR spectrophotometer was employed to identify the existence of different bioactive phytoconstituents, which further lead to reduction and stabilization of the ZnONP (Perkin-Elmer Spectrum 1000). FT-IR spectra of ZnOTP were obtained using KBr pellets in the wavelength range of 4500–400 cm^−1^ at room temperature.

#### Field emission scanning electron microscopy (FESEM)

Surface morphology and metal analysis of synthesized ZnOTP were determined with FESEM (Carl Zeiss, Germany) associated with energy dispersive X-ray spectroscopy (EDX-JEOL, JSM-5610). For FESEM analysis, dry sample powder was sprinkled over carbon tape and then coated with gold.

### Determination of total phenolic compounds

Involvement of phenolic compounds in aqueous TP extract (TPAE) and ZnOTP was determined by the Folin–Ciocalteu method as described by Mohamed *et al.*^27^ The results were calculated from the calibration curve of gallic acid standard solution and expressed as mg gallic acid equivalent (GAE) per g of extract. The experiment was repeated thrice.

### Determination of total flavonoid compounds

Total flavonoid content of TPAE and ZnOTP was detected by a colorimetric technique using aluminium chloride and expressed as mg rutin equivalent (RE) per g of extract from a standard rutin solution calibration curve.^28^ The experiment was repeated thrice.

### DPPH radical scavenging activity

Hydrogen or electron donating ability of TPAE and ZnOTP was determined by following the method with the help of a stable free radical, *i.e.* DPPH.^29^ Preparation of the reaction mixture was carried out by adding 150 μL of a 3.3 mM DPPH solution (prepared in methanol) to 100 μL of sample stock solutions made at different concentrations. After 30 minutes, each sample was analyzed spectrophotometrically at a wavelength of 517 nm. The potential of an antioxidant can be measured by the purple color bleaching reaction and its decrease in absorbance, calculated by the following formula:
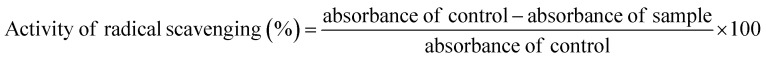


### *In vitro* anti-inflammatory activity

#### Membrane stabilization

The human red blood cell membrane stabilization method, with slight modification, was employed for determination of the anti-inflammatory activity of ZnOTP.^30^ Blood samples from healthy volunteers were collected after institutional ethical committee approval (IEC/16/365) and receiving informed written consent. It was also assured that they had not consumed NSAIDs two weeks prior to blood withdrawal. The collected blood samples were subjected to centrifugation for 15 minutes at 3000 rpm. The supernatant was discarded and the resulting pellet of the cells was thoroughly cleaned twice with normal saline solution (pH 7.0). Then, a 10% v/v suspension was prepared using normal saline solution. Various concentrations (25, 50, 100, 200 and 400 μg mL^−1^) of ZnOTP were prepared and 1 mL of blood suspension (freshly prepared) was added into each tube. For standard and control purposes, commercially available aspirin and normal saline were utilized, respectively. The resultant reaction mixture was incubated at 56 °C. After 30 min it was centrifuged for 5 min at 2500 rpm. The hemoglobin containing supernatant was scanned spectrophotometrically at a wavelength of 560 nm. The hemolysis percentage was assessed by assuming that the control resulted in complete hemolysis (100%). The calculation for the determination of percentage of human red blood cell membrane stabilization (% HRBCsMS) was performed using the following formula:
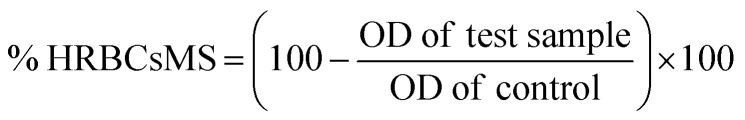


#### Determination of albumin denaturation

For the determination of albumin denaturation, the reaction mixture was prepared by adding 1% bovine albumin aqueous solution (Merck, Darmstadt, Germany) to different concentrations of ZnOTP, incubating for 20 min at 37 °C and then heating at 51 °C for 20 min. The reaction mixture was further analyzed for turbidity by scanning at 660 nm with a spectrophotometer using aspirin as a reference drug and distilled water as control.^31^ Denaturation caused by distilled water was considered as 100% denaturation. The percentage of protein denaturation protection was measured with the help of the following formula:^32^
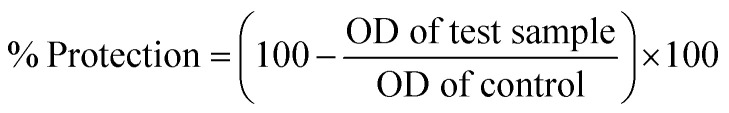


#### Proteinase inhibitor activity

To determine the level of trypsin inhibition, an assay mixture consisting of 0.06 mg of trypsin prepared by mixing in 1 mL of Tris–HCl buffer (20 mM, pH 7.4) was dissolved in each of the prepared concentrations of ZnOTP up to a volume of 2 mL and incubated at 37 °C for 5 min. Furthermore, 1 mL of 0.8% azocasein (prepared in 20 mM NaHCO_3_, pH 8.1) was added to the assay mixture and incubated for 20 min at 37 °C followed by mixing with 2 mL of (10% w/v) trichloroacetic acid. The resultant mixture was subjected to centrifugation at 12 000 rpm for 10 min, 2 mL of NaOH (1.0 M) was added to the supernatant and the mixture was scanned at 440 nm.^33^ Buffer was used as a blank. The inhibitory percentage of proteinase activity was analyzed by the given formula:
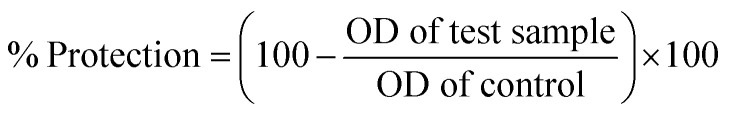


### Preparation of ZnOTP ointment

Formulations of ZnOTP were prepared utilizing a simple ointment base involving various ingredients, *i.e.* wool fat (25 g), hard paraffin (25 g), cetostearyl alcohol (25 g) and 425 g of white soft paraffin for each 500 g of the ointment. Hard paraffin and cetostearyl alcohol were mixed in required quantities with constant stirring at 60 °C in a water bath to achieve a gel-like consistency. Then, white soft paraffin as well as wool fat was mixed until it appeared as a homogenous mixture and it was allowed to cool. In the last step, prepared ZnOTP, at two different concentrations of 10 and 20 mg per gram of ointment, were added to the resultant homogenous ointment separately. A homogenizer was used for mixing, in order to obtain a uniform and smooth formulation.

### *Ex vivo* skin permeability determination

ZnOTP ointment was evaluated *ex vivo* for its ability to permeate through skin by employing rat skin. The experimental protocol for skin permeation was officially approved by the Institutional Animal Ethical Committee (IAEC) constituted in accordance with the guidelines of the Committee for the Purpose of Control and Supervision of Experiments on Animals (CPCSEA), Government of India.

The skin of the animal was shaved followed by careful elimination of subcutaneous fat and immediate cleaning. The static Franz diffusion method was used to analyze the percutaneous absorption study of the ZnOTP ointment.^34^ The receptor compartment was composed of 30 mL of physiological buffer solution equilibrated at 37 °C with the help of a thermostatic water jacket around the cell. The buffer solution filled in the diffusion cell was continuously agitated by a Teflon coated magnetic stirrer. A piece of rat skin was fixed between compartments, *i.e.* the donor and receptor, with a mean exposed area of about 3.14 cm^2^. Starting at zero time, 1 g of freshly prepared ZnOTP ointment was added into the exposed chamber of the Franz diffusion cell. 1 mL of the solution was taken out from the receptor compartment after defined time intervals such as 1, 2, 4, 6, 8, 10 and 24 h. The withdrawn sample was instantly replaced with an equal amount of prepared buffer solution. Samples were measured for Zn content using atomic absorption spectrometry.^35^

### Animals

A whole animal study was performed in accordance with the institutional guidelines of ethical conduct for purpose of use and care of research animals. The experimental protocol was acceptable according to the Institutional Animal Ethics Committee (Approval Number IAEC/SHIATS/PA16III/SEYAV09).

Forty-eight healthy adult male albino Wistar rats of about 180 ± 20 g were selected for the current *in vivo* study. Animals were housed in polypropylene cages in a well-ventilated animal house, and were allowed access to water *ad libitum* and a standard pellet food diet throughout the experimental work. Prior to the wounding day, for a one week period the experimental animals were subjected to acclimatization of the standard laboratory conditions [temperature (25 ± 2 °C), relative humidity (44–56%), and light and dark cycles (12 : 12 hours)].

### Acute dermal toxicity study

Dermal irritation and toxicity of ZnOTP were determined using a 200 mg kg^−1^ dose as per the Organization for Economic Cooperation and Development (OECD) guideline 402. The backs of the rats were shaved 24 h before the test. The test ointments were topically applied to the shaved backs and for the next 15 days the rats were observed regularly for adverse skin symptoms such as irritation, itching and redness, as well as inflammatory reactions and they were compared with a control group. Six rats in each group were utilized for the acute dermal toxicity study.

### Experimental protocol for dermal wound healing activity

Incision and excision wound models were employed to establish the wound healing activity of the ZnOTP ointment. The rats were randomly divided into four groups and each group consisted of six animals. The same grouping of rats was followed for another wound model.

Group 1 – Applied with ointment base

Group 2 – Applied with standard drug ointment, *i.e.* povidone iodine ointment USP (5% w/w)

Group 3 – Applied with 1% w/w ZnOTP ointment (ZnOTP1%)

Group 4 – Applied with 2% w/w ZnOTP ointment (ZnOTP2%)

Throughout the study, the respective drug treatments were applied topically once a day to all groups.

### Excision wound model

All rats from each group were shaved on their back region using a depilatory cream (Reckitt Benckiser Inc., UK). The next day, after administering anesthesia with mild ether, the rats were subjected to an excision wound on their shaved dorsal region. A circular outline of about 500 mm^2^ was drawn, and then the marked area was cut to a 2 mm thickness with a sterilized, sharp surgical blade and scissor.^36^ Normal saline dipped sterile cotton wipes were used for cleaning and the wounds were topically applied with their respective group treatment (the day of cutting was considered as day 0) until the wound was observed as being completely healed. During the whole experimental duration, the wounds were kept uncovered. Different parameters related to wound healing such as wound area, wound closure, epithelialization period and hydroxyproline content were analyzed.

#### Collection of blood and tissue

On the 17^th^ day (at the end of study), euthanasia was performed on the rats from each group of the excision wound model by cervical dislocation. Blood samples were collected from each group, preserved in collection tubes containing EDTA and utilized for determination of inflammatory markers. For various biochemical estimations and histochemical evaluation, skin samples from the healed regions were collected. For estimation of the antioxidant enzyme level, the preserved wound tissue samples were subjected to homogenization in TBS buffer, prepared by 50 mM Tris–HCl and 150 mM NaCl (1 : 2, pH 7.4) in an Ultra-Turax homogenizer. The homogenized samples were centrifuged at 5500 rpm for 15 min at 4 °C and the supernatants were preserved at −80 °C until further study.

### Determination of wound healing parameters

#### Wound closure rate and epithelialization

The wound closure rate was calculated by observing the wound area, through the regular tracing of the contour of the wound on transparent paper and placing it on a 1 mm^2^ graph paper sheet. Visual assessment of the wound was done by taking photographs of the wound area on every third day until complete healing of the wound was observed. The rate of wound closure was assessed by the following formula,^37^
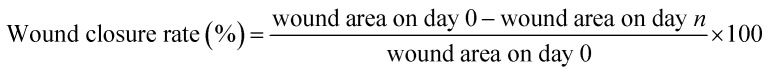
where *n* symbolizes the number of days (3^rd^, 6^th^, 9^th^, 12^th^, 15^th^ and 17^th^).

The epithelialization period is known as the number of days taken to remove the dead tissue from the wound without leaving any visible sign of the raw wound, and this important parameter was recorded.^38^

#### Hydroxyproline estimation

An estimation of the hydroxyproline content of the granulation tissue (collected from the excision wound model) was performed by cleaning the tissue with cold saline solution (0.9% w/v) and drying it at 60 °C. After drying, the granulation tissue samples were acid hydrolyzed with 6 N HCl for 4 hours at 130 °C, followed by the addition of 10 N sodium hydroxide for tissue hydrolysate neutralization to pH 7.0 and the samples were allowed to stand for 20 minutes to achieve chloramine-T oxidation. In the last step, perchloric acid (0.4 M) was poured into the reaction mixture and the color was observed by the addition of the Ehrlich reagent. The assay mixture was analyzed at 557 nm using a spectrophotometer (Shimadzu UV-1800, Japan). The content of hydroxyproline in the granulation tissue was estimated by a calibration curve of hydroxyproline and results were represented as mg g^−1^ of dry granulation tissue.^39^

#### Enzymatic antioxidant profile determination

The level of antioxidant enzyme activity in granulation tissue [superoxide dismutase (SOD), catalase (CAT) and glutathione peroxidase (GPx)] was estimated utilizing the supernatants which resulted from the wounded tissue homogenates.

SOD activity determination was performed by following the method described by Winterbourn *et al.*, which is associated with the prevention of NBT dye photoreduction by the SOD enzyme.^40^ Absorbency of the assay mixture was observed at a 560 nm wavelength and the activity of the SOD enzyme was expressed as unit per mg of protein in the granulation tissue. A unit of enzyme is considered as the concentration of enzyme responsible for inhibition of 50% of the oxidation reaction.

The CAT assay mixture was composed of the granulation tissue supernatant, 0.01 M phosphate buffer and 0.16 M H_2_O_2_. The resultant mixture was incubated for 1 min at 37 °C after mixing a dichromate : acetic acid reagent into it. The consequent sample was scanned at 570 nm.^41^ CAT activity was represented as μM of H_2_O_2_ consumed per mg protein in granulation tissue.

The GPx assay was analyzed according to the procedure of Flohé and Günzler, 1984, which depends on the rate of generation of 2-nitro-5-thiobenzoate (TNB) from DTNB linked with glutathione (GSH) oxidation due to the GPx enzyme.^42^ The absorbance of the reaction mixture was observed using a spectrophotometer at 412 nm and the result was expressed as μM mg^−1^ protein in tissue.

### Determination of inflammatory markers

Blood samples were collected in collecting tubes from the retro orbital vein of each animal from all groups at the end of the experimental work. *In vivo* estimation of inflammatory markers was performed by following the serum turbidimetric method of C-reactive protein estimation (CRP) utilizing a commercial kit (Beacon Diagnostics Ltd, India).

In addition to inflammatory markers, the level of fibrinogen in the plasma was analyzed by a colorimetric method.^43^ The reaction was initiated by the addition of 2.5% CaCl_2_ in plasma, leading to the generation of a clot-like structure. After washing the clot with distilled water, it was dissolved in 0.25 N NaOH in a water bath and then H_2_SO_4_ was added for the purpose of neutralization of the clot followed by mixing with the Folin–Ciocalteu reagent as well as 20% Na_2_CO_3_. The resultant assay mixture was incubated at 37 °C for half an hour and scanned at 620 nm.

### Histological study

Collected skin samples of the healed wound region were separately preserved in 10% formalin solution. The section for histochemical study was prepared by dehydration with alcohol followed by processing in xylene solution. Paraffin was used for blocking purposes, whilst 5 μm sections were cut and dyed with hematoxylin and eosin.^44^ The stained sections were viewed under a light microscope and photomicrographs of each group were saved.

### Incision wound model

Anesthesia was given to rats (pre-shaved) and a longitudinal line of 6 cm in length on either side of the vertebral column, parallel to the paravertebral area, was marked. An incision of 2 mm thickness was done using a sterile surgical scissor on the marked line.^45^ Normal saline dipped cotton swabs were used for attaining hemostasis and then the separated skin was stitched up by intermittent sutures at a distance of 1 cm with a curved needle (no. 11) and black silk surgical thread (no. 000). At each suture, the thread was carefully and strongly knotted on both ends of the wound to obtain a good closure. Wound stitches were left in the same condition for the next 24 hours and after that they were topically applied once a day with their respective treatment (regarded as day 1) until the wound was observed as being completely healed. On the 11^th^ day of post-wounding, the sutures were carefully removed from the skin, while the formulation was applied continuously until the 13^th^ day and the healed wound was further analyzed to determine its tensile strength by the constant water flow method.^46^

### Statistical evaluation

All data were expressed as mean ± standard error mean (SEM) of the six animals in each group and the results were compared statistically using one-way analysis of variance (ANOVA) followed by Dunnett’s test using the GraphPad Prism software. The mean values were considered statistically significant at *p* < 0.05.

## Results

### Characterization of ZnOTP

#### UV spectrophotometry

**Fig. 1:**
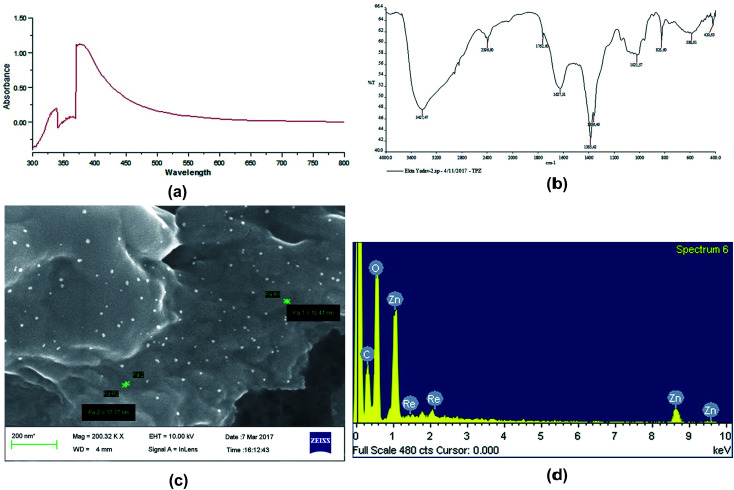
Characterization of green synthesized ZnOTP by (a) UV-Visible spectroscopy, (b) FT-IR spectroscopy, (c) FESEM microscopy and (d) EDX spectroscopy of the green synthesized ZnOTP.

Confirmation of the nanoscale of fabricated ZnOTP was carried out by obtaining an absorption spectrum of a sample using UV-Visible spectrophotometry. A surface plasmon resonance (SPR) absorption band occurred at 379 nm, which indicates absorption of ZnONP into the intrinsic band-gap as a result of electron transition to the conduction band from the valence band as shown in Fig. 1a. This highly blue-shifted absorption maximum band is an indication of nano-sized ZnOTP formation which is in good agreement with previous studies.^16^

#### FT-IR

FT-IR spectroscopy was performed to identify the role of active biomolecules of the plant extract in reduction and stabilization of ZnONP. The spectrum of ZnOPC exhibited peaks at 3427.47 and 2396.60 cm^−1^ (Fig. 1b) which were allotted to strong vibration absorption peaks of –OH stretching of the intramolecular hydrogen bond and the enol of 1,3-diketone, respectively.^47^ Peaks at 1762.60, 1627.51, 1385.42, 1359.46 and 1021 cm^−1^ represent the presence of C=C stretching of aromatic amine, (medium charge) vinyl, *cis*-tri substituted C=C stretching of aromatic amines, C–N stretching of aliphatic amines and C–H–OH stretching (typical for glucose residues of disaccharides). In addition, the spectrum showed bands at 588 and 420 cm^−1^ corresponding to metal–oxygen (M–O).^11,48,49^ Absorption bands between 400 and 600 cm^−1^ are characterized as Zn–O. Hence, the presented FT-IR peaks are concordant with reported work on the presence of rich amounts of secondary metabolites including phenolic compounds, flavonoids and alkaloids in ZnOTP.^24^

#### FESEM

The morphology of the surface of ZnOTP was viewed with the help of FESEM photomicrographs (Fig. 1c), which revealed a spherical structure with a size range of 10–20 nm of green synthesized ZnONPs at 200.32k× magnification power.

#### EDX

EDX spectra determined the metal composition of the nanostructured sample as shown in Fig. 1d, which shows the existence of strong peaks from zinc and oxygen atoms. Furthermore, a few other elements were also detected from weak signals in the EDX spectra such as carbon, phosphorous and rhenium. This confirms that secondary metabolites of TP were adsorbed on the surface of ZnONP as stabilizing agents.

### Total phenolic and total flavonoid content

Quantification of phenolic and flavonoid compounds in TPAE, as well as in ZnOTP, was done by a colorimetric method. The total phenolic content of green synthesized TPAE and ZnOTP was observed to be 15.2 ± 0.4 mg of GAE per g and 125.14 ± 1.21 mg of GAE per g, respectively. The bound flavonoid content of ZnOTP (3.56 ± 0.37 mg of RE per g) was found to be less, compared to that of TPAE (99.12 ± 1.10 mg of RE per g).

### Antioxidant activity

**Fig. 2:**
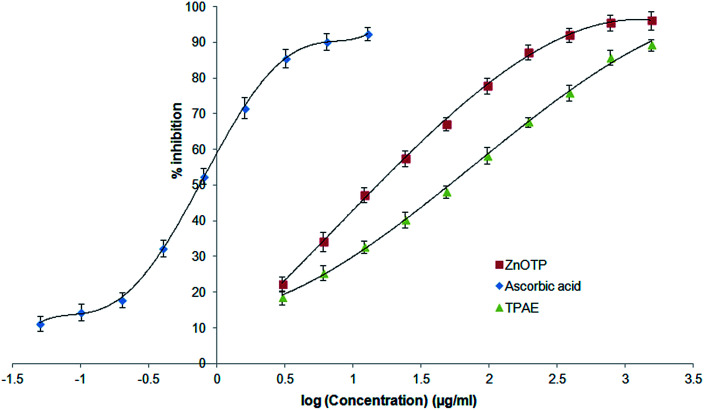
Comparative antioxidant activity of ZnOTP, TPAE and ascorbic acid by DPPH assay. Data expressed as mean ± SD (*n* = 3).

The ability of ZnOTP to scavenge free radicals was confirmed by a positive DPPH assay. ZnOTP exhibited significantly more inhibition potential than TPAE when compared to ascorbic acid (standard), as shown in Table 1. The green synthesized nanoparticles showed dose dependent DPPH scavenging activity. However, the results revealed that ZnOTP exhibited strong DPPH inhibition with an IC_50_ value of 15.16 ± 1.32 μg mL^−1^. Antioxidant activity of ZnOTP was found to be significantly higher than that of TPAE, as shown in Fig. 2, and this may be attributed to the conveyance of electron density positioned at the oxygen atom present in ZnO to the odd electron located at the nitrogen of DPPH, leading to a reduction in the intensity of the n → π* transition at a wavelength of 517 nm.^50^

**Table 1:** Antioxidant activity of TPAE, ZnOTP and ascorbic acid^a^

Sample	log IC_50_ (μg mL^−1^)	IC_50_ (μg mL^−1^)
TPAE	1.7069	50.92 ± 1.43*
ZnOTP	1.1808	15.16 ± 1.32*
Ascorbic acid	−0.1258	0.74 ± 0.07

a Each value is reported as the mean ± SD (*n* = 3). Statistical significance was at *p* < 0.05, *p < 0.05.

### *In vitro* anti-inflammatory assay of ZnOTP

At different concentrations of ZnOTP (25, 50, 100, 200, and 400 μg mL^−1^), *in vitro* anti-inflammatory activity was carried out by employing membrane stabilization and albumin denaturation along with proteinase inhibitory activity. Data showed significant membrane stabilization efficiency against human red blood cell membrane. Furthermore, at the highest concentration (400 μg mL^−1^), the protection rate of ZnOTP was observed to be similar to aspirin with a percentage inhibition of 78.4 ± 1.0% and 79.2 ± 0.4%, respectively. However, at 400 μg mL^−1^, ZnOTP effectively prevented denaturation of albumin with a maximum inhibition of 85.2 ± 1.1% when compared with aspirin which exhibited a 86.5 ± 0.6% maximum inhibition (Table 2).

**Table 2:** *In vitro* anti-inflammatory activity of ZnOTP and aspirin^a^

Treatment	Concentration (μg mL^−1^)	Membrane stabilization activity (%)	Albumin denaturation inhibition (%)	Proteinase inhibition (%)
Control	—	—	—	—
ZnOTP	25	25.2 ± 2.0*	22.3 ± 0.6*	30.1 ± 1.5*
ZnOTP	50	38.6 ± 1.1*	40.7 ± 1.1*	48.3 ± 0.7*
ZnOTP	100	49.4 ± 3.0*	59.4 ± 0.6*	60.2 ± 1.3*
ZnOTP	200	60.7 ± 2.3*	74.3 ± 1.3*	77.3 ± 0.5*
ZnOTP	400	78.4 ± 1.0*	85.2 ± 1.1*	88.4 ± 0.8*
Aspirin	400	79.2 ± 0.4*	86.5 ± 0.6*	86.8 ± 1.3*

a Each value is reported as the mean ± SD (*n* = 3). Statistical significance was at *p* < 0.05 when compared to the control group. *p < 0.05.

A proteinase inhibitory assay was also employed for confirmation of anti-inflammatory activity of ZnOTP. Slightly more prominent proteinase inhibitory activity was exhibited by ZnOTP compared to that of aspirin at 400 μg mL^−1^ concentration with maximum inhibition of 88.4 ± 0.8% and 86.8 ± 1.3%, respectively (Table 2).

### *Ex vivo* skin permeation and deposition studies

An *ex vivo* permeation study was performed to determine permeation of ZnOTP through the rat skin including the epidermis and dermis. This study shows a direct permeation comparison because the *stratum corneum* retains the property of permeability even after removal from the body. It was observed that the receptor compartment was composed of 0.11 μg cm^−2^ of Zn upon dermal exposure to the ZnOTP ointment which indicates very low penetration of Zn into the receptor compartment. After 24 h of ZnOTP treatment, the concentration of Zn deposited on the skin was found to be 6.1 μg cm^−2^.

### Acute dermal toxicity study

There was no sign of any dermal adverse reaction, *i.e.* irritation, erythema, swelling, itching, or mortality on the tested area of rat skin at the limit test dose level compared to the control group.

### Effect of ZnOTP on percentage wound closure and epithelialization period

**Fig. 3:**
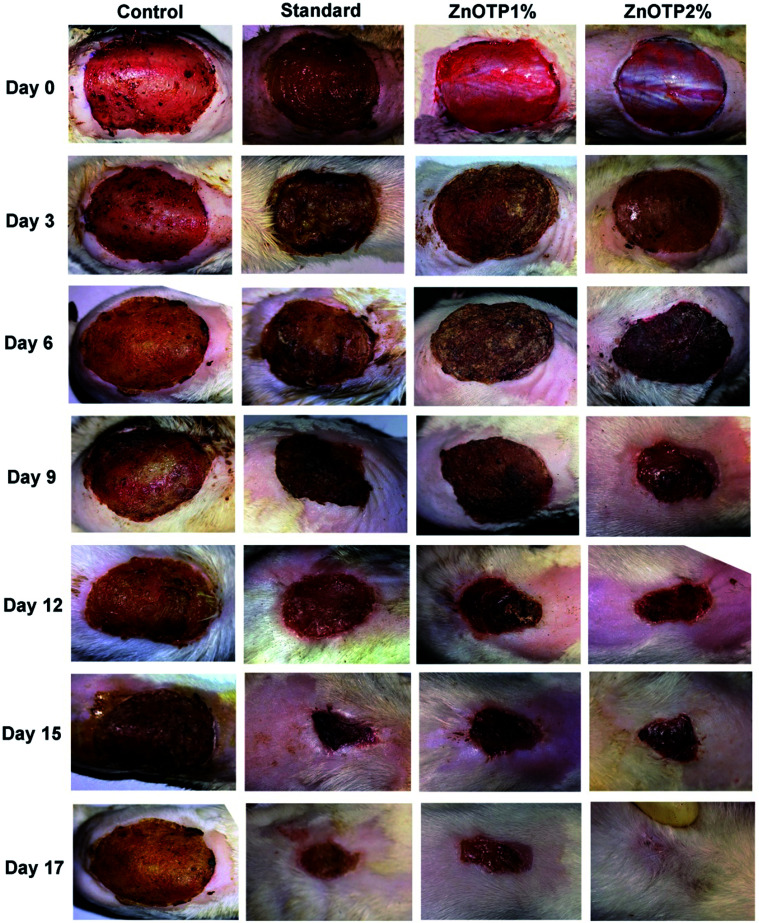
Photographic representation of the wound healing process in the excision wound model, showing the control (Group 1), standard drug (Group 2), ZnOTP1% treated (Group 3) and ZnOTP2% treated (Group 4) at 0, 3, 6, 9, 15 and 17 days post wounding.

**Fig. 4:**
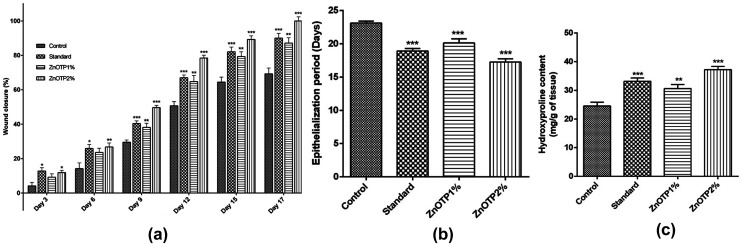
Effect of ZnOTP on excision wound expressed as (a) percentage of wound closure, (b) epithelialization period and (c) hydroxyproline content. Values are represented as the mean ± SEM (*n* = 6). Data were analyzed by one-way ANOVA followed by Dunnett’s test. Significant differences were *p < 0.05, **p < 0.01, and ***p < 0.001 in comparison to the control group.

On every third day, visible alterations in the area of the wound in the excision model were analyzed (Fig. 3). From the results, it was observed that on the 3^rd^ day after wounding, there was a significant reduction in the wound area of the standard group (*p* = 0.0100) as well as in the ZnOTP1% group (*p* = 0.0213) compared to the control group. However, on the 6^th^ day after wounding, ZnOTP at both dose levels (1% and 2% w/w) showed statistically significant (*p* = 0.0082 and *p* = 0.0420, respectively) wound closure compared to the control group. Furthermore, wound contraction percentage values of the ZnOTP2% group (26.77 ± 2.39) on the 6^th^ day were observed to be slightly higher than those of the standard group (25.89 ± 2.32). From day 9, the ZnOTP2% group (*p* < 0.0001) exhibited a significantly higher wound closure rate than the standard group (day 9, *p* = 0.0005; day 12, *p* = 0.0004; day 15, *p* = 0.0004; day 17, *p* = 0.0002) when compared to the control group. ZnOTP1% also showed a significant and dose dependant effect compared to the control group on days 9, 12, 15 and 17 with *p* values of 0.0046, 0.0016, 0.0020 and 0.0010, respectively (Fig. 4a).

In the case of the epithelialization period, the strongest effect was observed in the ZnOTP2% group which showed swift epithelialization followed by the standard and the ZnOTP1% group with 17.24 ± 0.5 (*p* < 0.0001), 18.91 ± 0.4 (*p* < 0.0001) and 20.12 ± 0.6 (*p* = 0.0007) days, respectively, when compared to the control group (23.12 ± 0.3 days), as shown in Fig. 4b.

### Hydroxyproline content

Animals treated with ZnOTP2% (*p* < 0.0001) showed higher hydroxyproline content than the standard group (*p* = 0.0003) when compared with the control group. Meanwhile, the ZnOTP1% group exhibited slightly less hydroxyproline content than the standard group and significantly higher content (*p* = 0.0074) than the control group (Fig. 4c).

### Enzymatic antioxidant profile of granulation tissue

**Fig. 5:**
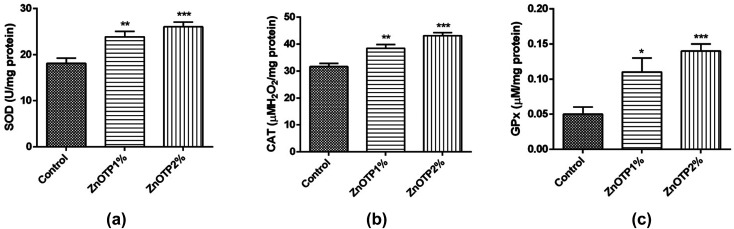
Effect of ZnOTP on the antioxidant enzyme profile in granulation tissue by estimation of the (a) SOD level, (b) CAT level and (c) GPx level. Data are represented as the mean ± SEM, and were analyzed by one-way ANOVA followed by Dunnett’s test. Significant differences were *p < 0.05, **p < 0.01, and ***p < 0.001 in comparison to the control group.

The tissue antioxidant enzymatic profile of the control group was observed less compared to other groups, which proved the condition of oxidative stress within the control group [Fig. 5a–c]. However, after treatment with the ZnOTP ointment (1 and 2% w/w), the SOD activity (*p* = 0.0045 and *p* = 0.0003, respectively), CAT activity (*p* = 0.0030 and *p* < 0.0001, respectively) and GPx activity (*p* = 0.0166 and *p* = 0.0008, respectively) were significantly accelerated in a dose dependent manner compared to the control group.

### Inflammatory markers

**Fig. 6:**
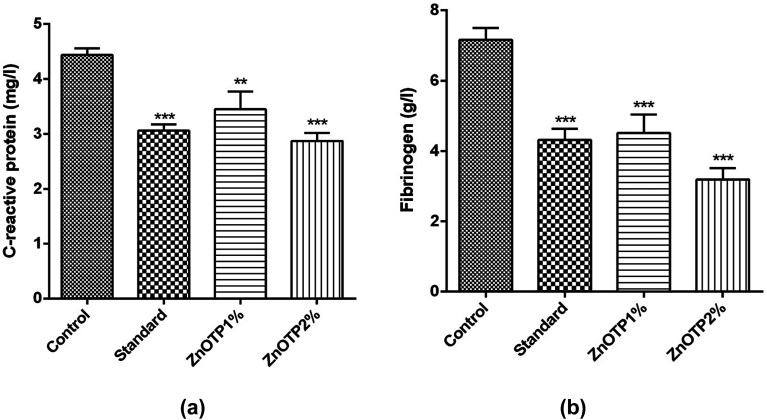
Effect of ZnOTP on the (a) CRP level and (b) fibrinogen level. Values are represented as the mean ± SEM (*n* = 6). Data were analyzed by one-way ANOVA followed by Dunnett’s test. The significant difference was ***p < 0.001 in comparison to the control group.

Levels of CRP and fibrinogen, *i.e.* well recognized inflammatory proteins, were examined to assess the inflammatory status of all groups. The elevation in concentration of both proteins was associated with the condition of inflammation. CRP content was observed to be significantly low at both dose levels in a dose dependent manner, *i.e.* ZnOTP2% (*p* < 0.0001) and ZnOTP1% (*p* = 0.0049), compared to that of the control group (Fig. 6a). However, the standard group showed a moderate effect on the CRP level compared with the control group. With regards to the data of the fibrinogen levels, these were found to be significantly reduced in the ZnOTP2% group (*p* < 0.0001) followed by the standard group (*p* = 0.0001) and the ZnOTP1% group (*p* = 0.0003), compared to the control group (Fig. 6b).

### Histopathological examination

**Fig. 7:**
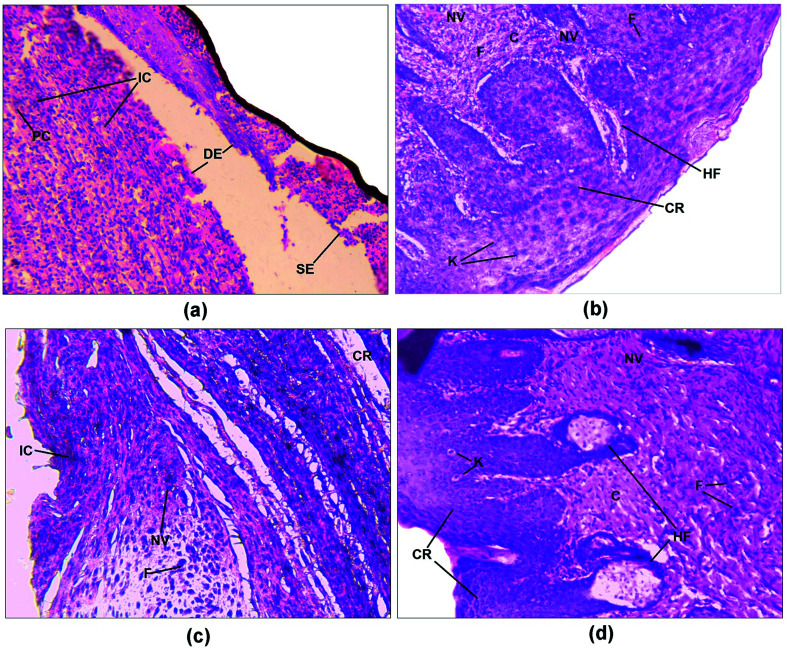
Photomicrographs (20×) of haematoxylin–eosin stained histological dermal sections of the wound areas for the (a) control group, (b) standard group, (c) ZnOTP1% group and (d) ZnOTP2% group. DE – disrupted epidermis; SE – separation of epidermis from dermis; IC – inflammatory cells; PC – pus cells; F – fibroblast; NV – neovascularization; C – collagen; CR – complete re-epithelialization; HF – hair follicle; K – keratinocytes.

Histopathological examination of the excision wound tissue was performed on day 17, as shown in Fig. 7a–d. The photomicrograph of the group treated with a simple ointment base exhibited a poor level of collagenation and abundant amounts of inflammatory cells, pus cells and fibroblasts as well as necrosis (Fig. 7a). The slide of the standard group exhibited less inflammatory cells compared to that of the control group along with complete re-epithelialization and elevated levels of collagen fibers, fibroblasts, keratinocytes, new blood vessels and hair follicles (Fig. 7b). In addition, sections of the ZnOTP1% group (Fig. 7c) showed considerable levels of collagen formation, fibroblast cells and signs of re-epithelialization, keratinization and less inflammatory cells compared to the control group. Sections of the ZnOTP2% group showed a state of complete tissue repair, indicated by re-epithelialization, rich collagen formation, a lack of inflammatory cells, neovascularization, hair follicles, keratinocytes and increased levels of fibroblasts (Fig. 7d) (Table 3).^3,51^

**Table 3:** Histological data of the granulation tissue obtained from different groups^a^

Groups	Re-epithelialization	Fibroblast	Collagenation	Neovascularisation	Inflammatory cells
Control	−	+	−	++	+++
Standard	+++	+++	+++	+++	−
ZnOTP1	++	++	++	++	++
ZnOTP2	+++	+++	+++	+++	−

a Haematoxylin and eosin stained wound skin sections were scored as −: absence, +: slight, ++: moderate and +++: extensive.

### Tensile strength

**Fig. 8:**
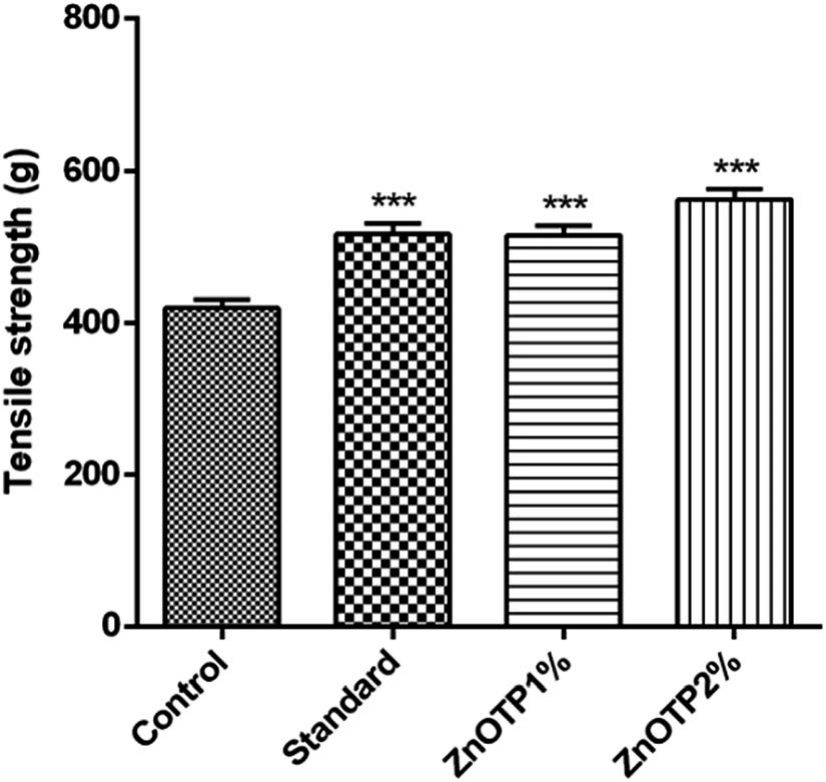
Effect of ZnOTP on tensile strength. Values are represented as the mean ± SEM (*n* = 6). Data were analyzed by one-way ANOVA followed by Dunnett’s test. The significant difference was ***p < 0.001 in comparison to the control group.

On the 13^th^ day, the incision wound model was employed for the tensile strength measurement of each group (Fig. 8). ZnOTP2% and the standard group exhibited higher breaking strength (*p* < 0.0001) followed by the ZnOTP1% group (*p* = 0.0001) compared to the control group.

## Discussion

Nowadays, the clinical treatment and management of wounds (moderate to severe) has been one of the biggest challenges in surgical dealings.^52^ A perfect wound repairing agent should have enough potential to facilitate the healing process as well as be devoid of adverse effects. Demands of new technologies that can accelerate the wound healing process along with the prevention of microbial invasion are at the forefront of research such as nanotechnology which has resulted in the ability to form green synthesized metal oxide nanoparticles mediated with plant extract. ZnONPs have long been used for their constitutional antimicrobial properties and the responsible mechanism is the inhibition of bacterial cellular metabolism by binding to the protein molecules resulting in the death of the microbes.^53^ There are a number of recent scientific reports that describe the antibacterial, anti-inflammatory and wound (acute as well as chronic) healing potential of ZnONP as well as TP.^54^

The current study is the first demonstration of the dermal wound healing potential of ZnOTP *via in vitro* as well as *in vivo* anti-inflammatory and antioxidant profile assays. The protective effect of ZnOTP against free radicals, detected with DPPH assay, has been attributed to the electrostatic attraction between negatively charged biologically active phytoconstituents (COO^−^, O^−^) and positively charged nanoparticles (ZnO = Zn^2+^ + O^2−^). ZnONPs attached with bioactive phytochemical aids increase their biological activity synergistically.^55^

Injured skin tissue is a possible vulnerable area for pathogenic microorganisms to enter into the blood and increase the severity of scalding through a dramatical increase in the activation of inflammatory mediators (α-chemokine and interleukin-8) which affect the function of fibroblasts and keratinocytes leading to boosted formation of collagen as well as maturation of granulation tissue.^56^ Considering this fact, utilization of an anti-inflammatory agent at the wound site to prevent invasion of microorganisms and to avoid infection, which ultimately supports an increase in the count of fibroblast cells and collagen formation, has become a valuable approach.^57,58^ The present study demonstrated that ZnOTP showed significant *in vitro* anti-inflammatory activity in a dose dependent manner by analyzing significant membrane stabilization and albumin denaturation along with proteinase inhibitory activity. It has been reported that ZnO nanoparticles upregulate TNF-α levels in acute as well as chronic inflammatory conditions and TNF-α, IL-1β and IL-6 coordinate inflammatory responses in immune cells and have been employed in the treatment of different inflammatory diseases.^59^ Assessment of membrane stabilization potential was performed under anti-inflammatory activity as there have been previous reports on the similarity between the erythrocyte membrane and lysosomal membrane.^60,61^ Considering the erythrocyte membrane stabilization effect, it can be predicted that ZnOTP may stabilize lysosomal membranes also. Anti-inflammatory efficiency of ZnOTP was also assured by another mechanism, *i.e.* inhibition of protein (albumin) denaturation. Protein denaturation is known to be a process involving the alteration of secondary and tertiary structure upon application which leads to a loss in their biological activities. As the concentration of ZnOTP increased, a considerable increase in albumin denaturation inhibition was observed and a similar pattern was followed by aspirin. Neutrophils of lysosyme are considered to be a great source of serine proteinase. It has been previously reported that the proteinase of leukocyte is responsible for the progression of tissue damage during inflammation; thus, proteinase inhibitors might play a pivotal role in tissue protection. Therefore, ZnOTP could be used as a potential therapeutic agent for the treatment of dermal wounds associated with an inflammatory reaction.

Since ZnOTP exhibited a significant *in vitro* antioxidant as well as anti-inflammatory effect, it was further utilized for evaluation of its wound healing potential. However, from the results of this investigation, it was observed that ZnOTP showed a strong dermal wound healing property in the rodent model which was well supported by the obtained information about various physical parameters (wound contraction rate, epithelialization period and tensile strength) along with biochemical (hydroxyproline content and antioxidant enzyme profile) and histological markers.

This study showed that treatment with ZnOTP2% significantly accelerated wound contraction rate in the early stages of the wound, *i.e.* from the 3^rd^ day after wounding, compared to the control group and even from day 6 to day 17 the wound area reduction was slightly higher than the standard group. As has been suggested in the literature, a faster wound contraction rate is associated with a reduction in the repairing time duration by decreasing the size of the wound as well as diminishing the extracellular matrix concentration which impedes the process of wound healing. In addition, wound contraction further aids re-epithelialization by managing seclusion of migrant keratinocytes.^26^ It may be hypothesized that topical application of ZnOTP might have directly promoted modulations of cytokines and growth factors which ultimately led to an increase in the rate of the wound repairing process as a result of rapid internalization in comparison to other groups.^36^

Epithelialization of the healed wound area in the ZnOTP2% applied group was observed at 17.24 ± 0.5 days, while in the standard treated group it was found to be 18.91 ± 0.4 days and partial epithelialization was noticed in the control treated animals. Formation of granulation tissue on the wounded area directs the re-epithelialization phase as a result of organization of epithelial cells around the newly formed tissue which act as a barrier between the wound region and the surrounding environment. The length of the wound healing period is considered an important parameter in wound healing activity evaluation. Wound re-epithelialization timing can be basically linked with the healing potential of the wound. It may be predicted that if re-epithelialization of injured tissue is rapid, faster regeneration will result in healing and *vice versa*.^62,63^ Thus, it might be established that ZnOTP more significantly reduces the period of epithelialization at both dose levels, which indicates an accelerated wound healing process.

Results of physical markers were further supported by biochemical estimations (hydroxyproline content and enzymatic antioxidant profile) of granulation tissue in groups treated with ZnOTP ointment. The hydroxyproline amount was directly linked with wound healing potential since it was proved to be one of the significant factors among three components of the triple helix collagen fibre.^64^ Therefore, a larger amount of hydroxyproline reflected significant turnover of collagen and indicated an accelerated rate of wound repairing.^65^ Topical administration of ZnOTP significantly increased the hydroxyproline content of the wound and collagen expression in the excision wound model.

Tensile strength has been considered as a marker of organization of collagen fibers subdermally in newly generated collagen.^66^ The quality and amount of collagen in epithelialized tissue, accelerated during the process of regeneration, direct the strength and level of wound repairing.^37^ Therefore, ZnOTP could be considered to act by this pathway to exhibit its wound healing activity as it showed significantly higher tensile strength compared to the control group and at a higher dose (2% w/w) than that of the standard group.

Oxidative stress due to over formation of ROS mediates cytotoxicity and interferes in the process of wound healing by introducing a detrimental effect on biomolecules such as DNA and proteins as well as lipid molecules. Scavenging of ROS could be a noteworthy approach to achieve the swift healing of wounds.^67^ Therefore, determination of the status of antioxidants (SOD, CAT and GPx) in healed tissue is also important because these enzymatic antioxidants accelerate the wound repairing process using devastating free radicals.^4^ The data of the present study revealed that ZnOTP exhibited an extensive increase in SOD, CAT and GPx antioxidant enzymatic activity. It has been demonstrated that an augmented level of SOD leads to the conversion of hydrogen peroxide (H_2_O_2_) from superoxide which is further employed for neutralization into water with the help of lysosomal CAT or by mitochondrial GPx and leads to reduction of oxidative stress which is thought to accelerate healing of wounds by promoting various phases of reparation.^68^ Our results regarding an increase in the antioxidant enzyme profile by ZnOTP2% is superior to the standard group which may be assigned to the antioxidant effect of ZnOTP by decreasing oxidative stress in the wound area due to the existence of bioactive molecules such as phenolic and flavonoid compounds, ultimately causing a synergistic effect in wound repairing.^69^

All the above described parameters were ultimately confirmed by the observation of cellular characteristics of granulation tissue with the help of a histopathological study. The appearance of fibroblasts, complete re-epithelialization, neovascularization, and fewer inflammatory cells were observed in the photomicrographs (Fig. 7) of the tissue obtained from the ZnOTP treated group, which formed the basis for the elevation of collagenation at the wound site.^70^

An acute wound results in a wound bed to be healed by removing the necrotic tissue, debris, and bacterial contaminants as well as recruiting and activating fibroblasts. Under normal conditions inflammation is a self-limiting process. Excessive inflammation, however, limits wound healing.^71^ Therefore, ZnOTP was further employed to assess *in vivo* anti-inflammatory activity by CRP and fibrinogen content determination. An acute phase family of proteins includes CRP which is released from hepatocytes and its concentration increases significantly in response to interleukin-1 and 6 (monocyte origin mediators) as a result of infection, tissue damage and inflammatory diseases.^72^ The ZnOTP applied group exhibited a reduced amount of plasma inflammatory content compared to the control group. The results concluded that ZnO as well as polyphenolic compounds of TP might have contributed to the anti-inflammatory response and ultimately led to faster healing of the acute wound.^26,73,74^ However, further future research is required to corroborate this claim.

With regard to the toxicity of ZnO, permeation studies demonstrated that with the ZnOTP ointment, a major quantity of ZnO nanoparticles remained localized within the *stratum corneum* while a lower concentration penetrated into the receptor compartment. It was suggested that, due to the localizing effect in the skin, ZnO is favorable for the purpose of topical application with minimum systemic toxicity.^75^ In the case of oral administration of ZnONP (100 nm), a dose greater than 125 mg kg^−1^ was suggested as being significantly toxic in rats.^76^ Furthermore, no adverse dermal reaction was observed throughout the dermal toxicity study, hence it was believed to be safe at a 200 mg kg^−1^ dose.

## Conclusion

ZnOTP were biosynthesized and explored for their effect on the wound healing process using excision and incision models. ZnOTP exhibited SPR at 379 nm with an average 10–20 nm particle size. FTIR studies confirmed the involvement of bioactive phytoconstituents in the capping of ZnONP during the reduction process. These green synthesized nanoparticles showed strong *in vitro* antioxidant and anti-inflammatory activity. Topical treatment with ZnOTP over the injured dermal tissue resulted in quick formation and deposition of collagen fibers, tissue granulation and reconstruction of epithelial lining due to a reduction in oxidative stress and inflammatory reactions which led to rapid closures and healing of the wound, compared with the control group, at both dose levels using the Wistar albino rat model. The current investigation further untangles other beneficial insights into the characteristic dermal wound repairing attributes of ZnOTP and uncovers new scope for clinical scientists in the development and design of modern therapeutic means using novel nano-ointment formulations.

## Conflicts of interest

The authors declare no conflict of interests.

## Supplementary Material
