# Expression of concern: Ameliorative effect of biofabricated ZnO nanoparticles of *Trianthema portulacastrum* Linn. on dermal wounds *via* removal of oxidative stress and inflammation

**DOI:** 10.1039/d3ra90011h

**Published:** 2023-02-03

**Authors:** Ekta Yadav, Deepika Singh, Pankajkumar Yadav, Amita Verma

**Affiliations:** a Bioorganic & Medicinal Chemistry Research Laboratory, Department of Pharmaceutical Sciences, Sam Higginbottom University of Agriculture, Technology & Sciences (SHUATS) Allahabad 211007 India amitaverma.dr@gmail.com; b Pharmaceutics Laboratory, Department of Pharmaceutical Sciences, Sam Higginbottom University of Agriculture, Technology & Sciences (SHUATS) Allahabad-211007 India pypharm@gmail.com

## Abstract

Expression of concern for ‘Ameliorative effect of biofabricated ZnO nanoparticles of *Trianthema portulacastrum* Linn. on dermal wounds *via* removal of oxidative stress and inflammation’ by Ekta Yadav *et al.*, *RSC Adv.*, 2018, **8**, 21621–21635, https://doi.org/10.1039/C8RA03500H.

*RSC Advances* is publishing this expression of concern in order to alert our readers that we are presently unsure of the reliability of the data reported in Fig. 3 of the article. A number of the wound healing images in Fig. 3 have been duplicated either within the figure, or in two of the authors' publications in *Biomedicine & Pharmacotherapy*.^1,2^

The authors have provided a replacement figure for consideration and say that the new data does not affect the conclusions of the paper. The Royal Society of Chemistry has asked the affiliated institution to investigate this matter and establish whether the replacement images provided by the authors provide an accurate representation of the experiments that were conducted, and confirm the integrity and reliability of the new data provided. An expression of concern will continue to be associated with this manuscript until we receive information from the institution on this matter.

Laura Fisher

24^th^ January 2023

Executive Editor, *RSC Advances*

## Supplementary Material
