# Correction and removal of expression of concern: Ameliorative effect of biofabricated ZnO nanoparticles of *Trianthema portulacastrum* Linn. on dermal wounds *via* removal of oxidative stress and inflammation

**DOI:** 10.1039/d3ra90056h

**Published:** 2023-06-28

**Authors:** Ekta Yadav, Deepika Singh, Pankajkumar Yadav, Amita Verma

**Affiliations:** a Bioorganic & Medicinal Chemistry Research Laboratory, Department of Pharmaceutical Sciences, Sam Higginbottom University of Agriculture, Technology & Sciences (SHUATS) Allahabad 211007 India amitaverma.dr@gmail.com; b Pharmaceutics Laboratory, Department of Pharmaceutical Sciences, Sam Higginbottom University of Agriculture, Technology & Sciences (SHUATS) Allahabad-211007 India pypharm@gmail.com

## Abstract

Correction and removal of expression of concern for ‘Ameliorative effect of biofabricated ZnO nanoparticles of *Trianthema portulacastrum* Linn. on dermal wounds *via* removal of oxidative stress and inflammation’ by Ekta Yadav *et al.*, *RSC Adv.*, 2018, **8**, 21621–21635, https://doi.org/10.1039/C8RA03500H.

The authors regret that there was an error in Fig. 3 whereby incorrect wound healing images were used. This was due to poor management of a large dataset of photographs and we apologize for this purely unintentional error. The correct Fig. 3 is provided herein. The accuracy and integrity of the replacement images in Fig. 3 have been confirmed by an investigation from the affiliated institution.

**Fig. 3:**
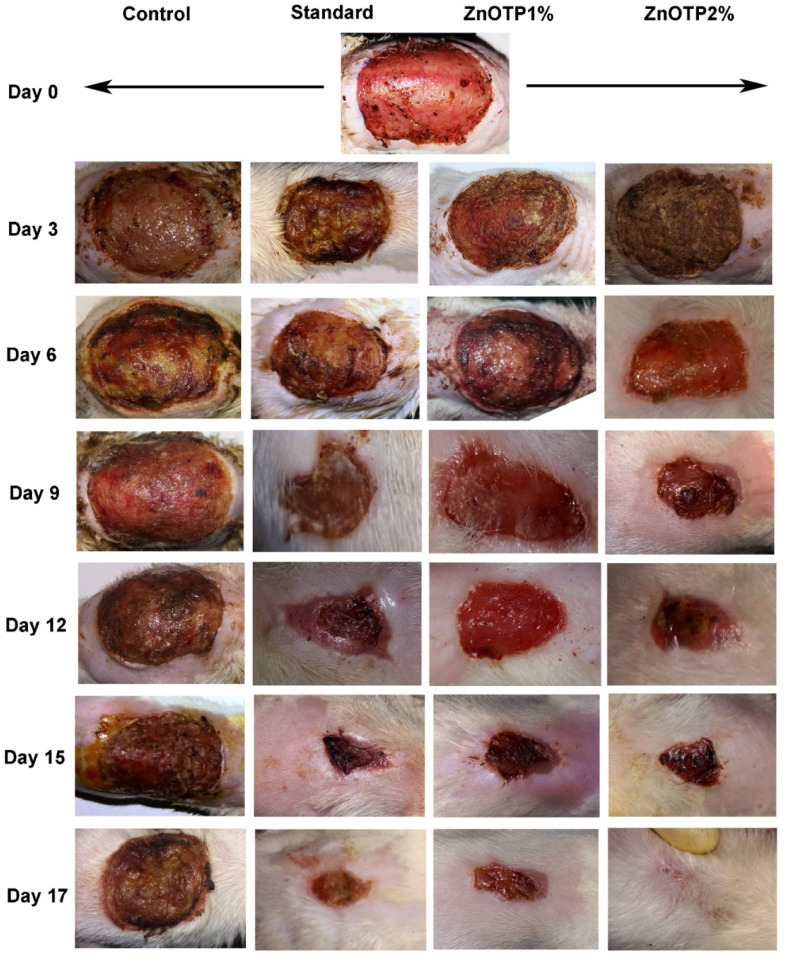
Photographic representation of the wound healing process in the excision wound model, showing the control (Group 1), standard drug (Group 2), ZnOTP1% treated (Group 3) and ZnOTP2% treated (Group 4) at 0, 3, 6, 9, 12, 15 and 17 days post wounding.

This correction supersedes the information provided in the Expression of Concern related to this article.

The Royal Society of Chemistry apologises for these errors and any consequent inconvenience to authors and readers.

## Supplementary Material

